# Caller Volume and Gestational Length at an Abortion Fund After *Dobbs*

**DOI:** 10.1001/jamanetworkopen.2025.46508

**Published:** 2025-12-03

**Authors:** Katrina Kimport, Rosalyn Schroeder, Corinne H. Rocca

**Affiliations:** 1 University of California, San Francisco, School of Medicine, Department of Obstetrics, Gynecology and Reproductive Sciences, Advancing New Standards in Reproductive Health, Oakland

## Abstract

**Importance:**

Since the Supreme Court’s 2022 decision in *Dobbs v Jackson Women’s Health Organization,* state legislatures have enacted laws severely restricting abortion. Facility case studies have reported post-*Dobbs* increases in patient volume and gestational length at abortion.

**Objective:**

To understand changes from before to after the *Dobbs* decision in the overall volume of callers and gestational length of their pregnancies at a large, regional abortion fund.

**Design, Setting, and Participants:**

This cross-sectional study used data from monthly caller records (June 2016-June 2024) from the District of Columbia Abortion Fund (DCAF) using interrupted time series analyses with segmented regression. DCAF serves the Washington, DC, area, which is unique in its absence of gestational restrictions, service availability, and proximity to states with post-*Dobbs* restrictions. Data were analyzed from November 2024 through August 2025.

**Exposure:**

Time in months, with change points and discontinuities after the *Dobbs* decision. To contextualize trends, change points and discontinuities were examined at the onset of the COVID-19 pandemic, the enactment of a 6-week abortion ban in Texas, and the enactment of a 12-week ban in North Carolina.

**Main Outcomes and Measures:**

Overall call volume and gestational length of callers, measured dichotomously (first trimester vs ≥13 weeks) and continuously (weeks’ gestation).

**Results:**

Among 43 351 DCAF caller records, 31% had pregnancies at 13 weeks’ gestation or greater. After the *Dobbs* decision, there was a sudden and substantial reversal of a prior 6-year trend of increasing call volume, as indicated by both an immediate drop (from 721 to 663 callers per month; coefficient, −10.7% [95% CI, −16.8% to −4.7%]), and a continued decrease over the subsequent year (coefficient, −12.7% [95% CI, −13.6% to −11.6%] per month). Concurrently, there was a sharp increase in the proportion of callers whose pregnancies were at 13 weeks’ gestation or later, from approximately 22% just before *Dobbs* to 32% the month after the *Dobbs* decision (odds ratio [OR], 1.61 [95% CI, 1.39 to 1.87]); this increase continued (OR per month, 1.07 [95% CI, 1.06 to 1.09]), reaching a peak of 53% per year post-*Dobbs*. Both trends reversed after the enactment of North Carolina’s 12-week ban.

**Conclusions and Relevance:**

In this cross-sectional study of caller records at a large abortion fund, there were immediate and lasting changes in the volume and gestational length of callers following the *Dobbs* decision, suggesting differential redistribution of access or care-seeking by trimester and/or delays in presentation for care.

## Key Points

**Question:**

Did call volume and gestational distribution of callers to a large regional abortion fund change after the *Dobbs v Jackson Women’s Health Organization *decision overturning the constitutional right to abortion?

**Findings:**

This cross-sectional study including using monthly caller data from 43 351 calls from 2016 to 2024 found a decrease in call volume after the *Dobbs* decision and an increase in the proportion of callers whose pregnancies were at 13 weeks or later (estimated 22% before vs 32% the month after *Dobbs*, reaching a peak of 53% 1 year after *Dobbs)*.

**Meaning:**

This cross-sectional study found that after *Dobbs,* there were immediate and lasting changes in the volume and gestational distribution of callers at a large regional abortion fund.

## Teaser

This cross-sectional study examines changes in the volume and gestational length of callers to a large regional abortion fund before and after the *Dobbs v Jackson Women’s Health Organization *decision.

## Introduction

In the month following the US Supreme Court’s 2022 *Dobbs v. Jackson Women’s Health Organization* (hereafter, *Dobbs*) decision overturning the constitutional right to abortion, 9 states enacted total abortion bans, 3 banned abortion after 6 weeks’ gestation, and 3 others banned abortion after 15 or 18 weeks’ gestation. Additional states followed, such that, 1 year after the decision, more than one-third of states had enacted limits on abortion that were earlier than allowable pre-*Dobbs*.^1^ These restrictions were followed by substantial reductions in the number of facility-based abortions in states where abortion was newly restricted and substantial increases in the number of facility-based abortions in states where abortion remained broadly legal,^2^ as well as higher than expected infant mortality in ban states.^3^ However, these laws were not followed by a reduction in the number of abortions; rather, the number of abortions increased following *Dobbs*, potentially in part because of an increase in funding support available to abortion seekers.^4,5^

Existing studies tracking abortion incidence and geographical distribution do not comprehensively track gestational length at abortion, limiting understanding about whether and to what extent post-*Dobbs* restrictions have affected the gestational distribution of abortions. Two case studies of facilities in the US Northwest found increases in both volume of patients seeking abortions and gestational length at abortion post-*Dobbs*,^6,7^ consistent with pre-*Dobbs* research finding that policy-related barriers to abortion delay people seeking abortion and result in later presentation for care.^8,9,10^ This study adds to the emerging literature through an examination of callers to a large, geographically unique, regional abortion fund: the District of Columbia Abortion Fund (DCAF).

Abortion funds are nonprofit organizations that offer financial support to offset the costs of abortion, and they have long played a key role in helping people seeking abortion surmount the multiple barriers they face in accessing abortion care.^11,12,13,14,15^ Thus, abortion fund callers provide a window into a region’s population of people seeking abortion at any given time. Staffed primarily by volunteer case managers, DCAF provides pledges, paid directly to abortion facilities, to cover part or all of the cost of callers’ clinical abortion care. Callers need to have an appointment before DCAF can make a pledge for their abortion and are typically referred to DCAF by the clinician or clinic providing the abortion. DCAF serves people seeking abortion from the greater District of Columbia (DC) area (ie, DC and surrounding areas in Maryland and Virginia), wherever they may be seeking care (ie, locally or elsewhere in the US), and people from elsewhere seeking abortion who travel to the greater DC area for care. In 2022 alone, DCAF pledged $2.3 million to support more than 7000 callers’ abortion care.

In addition to its size, DCAF’s geographic location makes it an appropriate case study for examining potential effects of *Dobbs* on the gestational distribution of people seeking abortion. The states that banned or severely restricted abortion post-*Dobbs* were concentrated in the US South.^16^ However, abortion remained broadly legal in DC and the neighboring states of Maryland and Virginia. People seeking abortion from many of the Southern states, where abortion was banned or severely restricted, could travel to the DC area by car more easily than to other states where abortion remained broadly legal. Additionally, the DC region is unique in its abortion service availability: there is no gestational limit in DC or Maryland, and there are multiple local health care facilities that offer care throughout pregnancy. The breadth of this care availability enables observation of changes in gestational distribution.

This study uses more than 8 years of DCAF caller records, including 2 years of post-*Dobbs *data, to analyze changes, if any, in the volume and gestational length of callers over time. Our hypotheses, in line with findings from the facility case studies,^6,7^ were that the volume and proportion of callers seeking funding for abortion care at 13 weeks’ gestation or greater increased after the *Dobbs* decision, reflected by both an immediate jump and subsequent increases over time post-*Dobbs*.

## Methods

This cross-sectional study was reviewed and approved by the University of California, San Francisco, institutional review board, which determined informed consent was not required because the data were deidentified. This study is reported following the Strengthening the Reporting of Observational Studies in Epidemiology (STROBE) reporting guideline for cross-sectional studies.

### Measures

DCAF provided the study team with a deidentified dataset of callers from June 2016 through June 2024 (97 months). During this period, DCAF’s volunteer case managers used an online platform for tracking callers, which required they log every call and record information from the call during or immediately afterwards. Each entry in the deidentified dataset represented 1 pregnancy and included intake date (captured by month and year), caller’s self-reported gestational length at the time of intake (captured in weeks), and caller’s state of residence. Using US Census Bureau designations, we categorized callers into a geographic region based on their state of residence; callers from the South were further divided into local (from DC, Virginia, or Maryland) vs nonlocal.

Our outcomes of interest were total DCAF call volume and gestational length of pregnancies at the time of the first call. Because abortion restrictions often differentiate between first trimester vs later abortions, and because abortion method options differ between these groupings, we first treated gestational length as a binary variable (<13 vs ≥13 weeks’ gestation). We repeated analyses treating gestational length continuously (in weeks). The independent variable was calendar time in months from June 2016 through June 2024.

Because our primary interest related to changes in outcomes at and after the *Dobbs* decision (in effect June 24, 2022), we included a spline or interruption at July 1, 2022 (chosen because data were available by calendar month, so we placed splines and interruptions at the month juncture subsequent to the event). We also identified a priori other significant events we hypothesized could have led to changes in call volume or caller gestational length to consider for inclusion in models to appropriately contextualize *Dobbs* effects. These events included the onset of the COVID-19 pandemic (March 16, 2020; modeled April 1, 2020), which had sweeping effects on reproductive health care access and pregnancy preferences,^17,18^ and the enactment of 2 state-level abortion policies. First, we examined the enactment of Senate Bill 8 (SB8) in Texas, which banned abortion after cardiac activity is detectible, ie, approximately 6 weeks after last menstrual period (September 1, 2021; modeled October 1, 2021). Texas is the second most populous US state, and although Texas residents would likely seek abortion care from geographically closer states, research has found evidence of Texans traveling to the greater DC area for abortion services.^19,20^ Second, we examined the enactment of a 12-week abortion limit in North Carolina (July 1, 2023; modeled August 1, 2023), due to the removal of care availability later in pregnancy from a state that, during the first year after *Dobbs,* experienced a surge in abortions compared to pre-*Dobbs*.^21^ We did not include the May 1, 2024, enactment of a 6-week ban in Florida, which we would have modeled as June 1, 2024, as it was only 1 month from the end of our study period.

### Statistical Analysis

Data analysis was performed from November 2024 through August 2025. Among 45 617 unique DCAF caller records between June 2016 and June 2024, 178 (0.4%) were missing data on month of call and 2088 (4.6%) were missing the caller’s gestational length, leaving an analysis sample of 43 351 observations (95.0%) (eFigure in Supplement 1).

We conducted segmented regression analysis of interrupted time series data to examine trajectories of DCAF call volume, trimester, and gestational length from June 2016 through June 2024.^22,23^ Our modeling approach was linear regression (for call volume and gestational length) and logistic regression (for trimester), with calendar month modeled as a linear spline with knot locations at each of the 4 events (to capture slope changes) and with discontinuities at the knots (to capture jumps or level changes). We confirmed that we had not missed notable change points by using data visualization and examining Harrell recommended knot locations.^24^ For each outcome, we first included all 4 change points and discontinuities (jumps) in outcomes at the change points; we then removed discontinuities at which substantial changes in overall levels or slopes of outcomes were not detected. Given our large sample size that resulted in highly precise estimates, we used a 2-sided *P* < .01 significance level. The final call volume model excluded the SB8 change point and discontinuity. The final gestation models excluded the SB8 discontinuity. To account for minor evidence of seasonality of call volume (lower at the end of each year, higher at the beginning of each year), we included annual quarter in the call volume model.^18,25^

We conducted 3 sensitivity analyses. To examine whether results were sensitive to the precise placement of the *Dobbs* interruption, we repeated analyses, moving it to June 1, 2022. Second, given the nonnormal distribution of gestational lengths, we repeated continuous gestation analyses examining median rather than mean gestation. Finally, to determine whether results were sensitive to missing gestational length data for 2088 callers (4.6%), we repeated analyses assuming all these callers were at 4 and then 18 weeks’ gestation. Analyses were conducted with Stata statistical software version 18 (StataCorp).

## Results

Among 43 351 calls to DCAF between June 2016 and June 2024, approximately two-thirds (68.0%) were from the local region (DC, Virginia, or Maryland). Another 18.5% were from the US South outside the local region, 2.8% from the Northeast, 1.3% from the Midwest, 0.3% from the West, and 9.0% unknown or missing. Overall, 68.8% of callers’ pregnancies over the study period were in the first trimester and 31.2% were after the first trimester. Mean (SD) gestational length was 11.2 (7.0) weeks with a strong right skew (median [IQR], 8 [6-15] weeks).

### Call Volume

**Figure 1.**
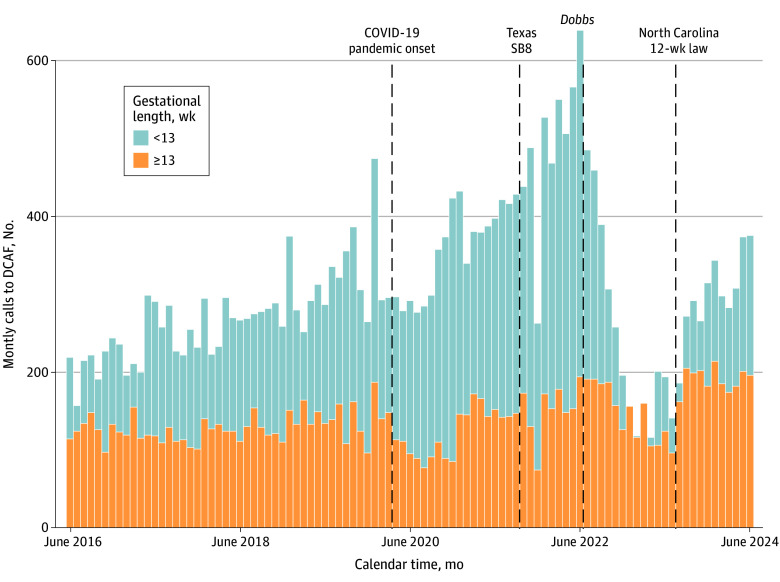
District of Columbia Abortion Fund (DCAF) Call Volume and Major Events Associated With Abortion Access Call volume stratified by caller gestational length, with those with less than 13 weeks’ gestation overlaid with those with 13 weeks’ gestation or greater. Low call volume in December 2021 is due to the abortion fund being closed for 2 weeks that month. SB8 indicates Texas Senate Bill 8.

**Figure 2.**
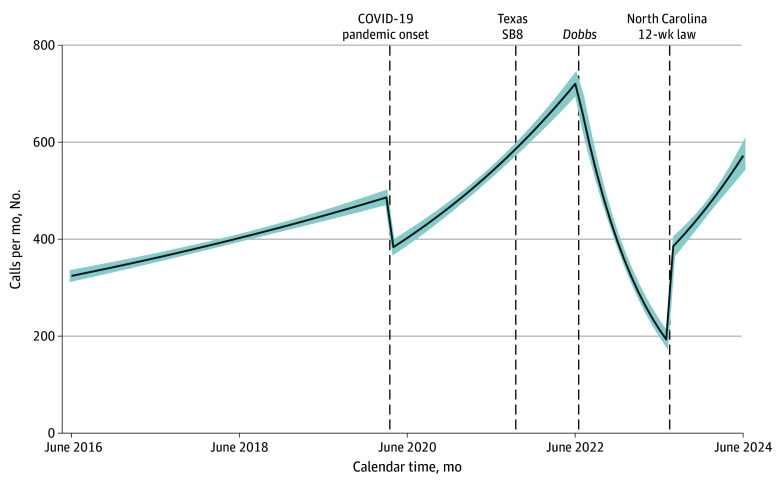
Estimated District of Columbia Abortion Fund (DCAF) Call Volume and Major Events Associated With Abortion Access Based on Segmented Regression Analysis of Interrupted Time Series Data SB8 indicates Texas Senate Bill 8.

Overall call volume to DCAF declined precipitously after the *Dobbs* decision. Specifically, over the 2 years prior to *Dobbs*, call volume increased 2.4% (95% CI, 2.2% to 2.6%) each month (mean [range], 13 [9 to 17] additional callers every month) (Figure 1, Figure 2, and Table 1). After *Dobbs*, call volume dropped immediately, from a high of an estimated 721 calls the month prior to *Dobbs* to 663 the month following. Volume continued to decrease by 10.2% (95% CI, −11.1% to −9.3%) per month (mean [range], −39 [−65 to −21] fewer callers every month) until North Carolina’s 12-week law was enacted, when it increased in 1 month by 192 callers and increased again by a mean (range) of 19 (16 to 22) callers per month thereafter.

**Table 1.** Segmented Regression Models of Interrupted Time Series Data for Changes in the Volume of Calls to the District of Columbia Abortion Fund, June 2016 to June 2024

Measure	% (95% CI)		Mean change per mo, No.	*P* value	
	Coefficient (95% CI)	Slope^a^		Difference from zero	Change from prior slope
Volume of callers					
Continuous (since June 2016), per mo	0.9 (0.8 to 1.0)	0.9 (0.8 to 1.0)	4^b^	<.001	NA
COVID-19					
Incident (April 2020)	−24.5 (−29.0 to −20.0)	NA	−103^c^	<.001	NA
Continuous after COVID-19 onset, per mo	1.5 (1.3 to 1.8)	2.4 (2.2 to 2.6)	13^b^	<.001	<.001
*Dobbs* decision					
Incident (July 2022)	10.7 (−16.8 to −4.7)	NA	−58^c^	<.001	NA
Continuous after *Dobbs* decision, per mo	−12.7 (−13.6 to −11.6)	−10.2 (−11.1 to −9.3)	−39^b^	<.001	<.001
12-wk law change in North Carolina					
Incident (August 2023)	79.1 (69.4 to 88.8)	NA	192^c^	<.001	NA
Continuous after 12-wk law, per mo	14.2 (13.0 to 15.4)	3.9 (3.1 to 4.8)	19^b^	<.001	<.001
**Season**					
January-March	0 [Reference]	NA	NA	NA	NA
April-June	0.7 (−0.2 to 0.3)	NA	NA	.61	NA
July-September	−3.2 (−6.1 to −2.6)	NA	NA	.03	NA
October-December	−5.7 (−8.5 to −3.0)	NA	NA	<.001	NA

Abbreviations: *Dobbs*, *Dobbs v Jackson Women’s Health Organization* decision; NA, not applicable.

^a^ Slope between change points can be derived by summing β coefficients.

^b^ Change in callers per month.

^c^ Change in callers that month.

### Trimester and Gestational Length

**Figure 3.**
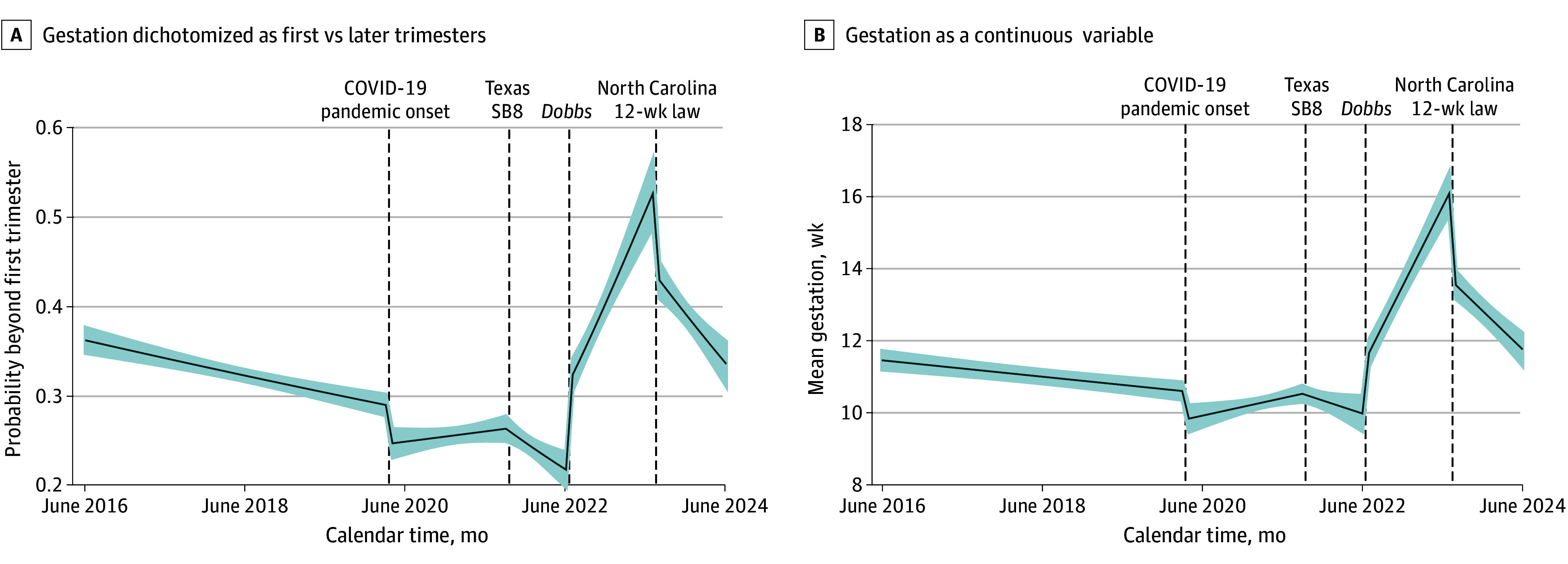
Estimated Trimester and Gestational Length of Pregnancies of Callers to the District of Columbia Abortion Fund (DCAF) and Major Events Associated With Abortion Access Based on Segmented Regression Analysis of Interrupted Time Series Data

Over the months preceding the *Dobbs* decision, the estimated percentage of callers who were beyond the first trimester decreased from approximately 26% the month after SB8 to approximately 22% (odds ratio [OR], 0.97 [95% CI, 0.96 to 0.99]) (Figure 3 and Table 2). After *Dobbs*, the estimated percentage increased dramatically to 32% the first month and continued increasing approximately 1.6 to 1.8 percentage points per month (OR, 1.07 [95% CI, 1.06 to 1.09]), reaching a peak of 53% a year later. With the enactment of North Carolina’s 12-week law, this trend reversed: there was a sharp drop in the proportion of callers seeking later abortions the month after the 12-week law (to an estimated 43%), and the proportion of callers requesting abortions at 13 weeks’ gestation or later declined approximately 0.9 percentage points per month thereafter (OR, 0.96 [95% CI, 0.94 to 0.98]).

**Table 2.** Segmented Regression Models of Interrupted Time Series Data of Changes in the Trimester and Gestational Length of Pregnancies of District of Columbia Abortion Fund Callers, June 2016 to June 2024

Measure	Coefficient (95% CI)	OR (95% CI)	Slope^a^	Monthly change, mean	*P* value, difference from zero	*P* value, change from prior slope
**Trimester**						
Continuous (since June 2016), per mo	NA	0.99 (0.99 to 0.99)	0.99 (0.99 to 0.99)	−1.6^b^	<.001	NA
COVID-19 onset						
Incident (April 2020)	NA	0.80 (0.71 to 0.89)	NA	−4.3^c^	<.001	NA
Continuous after COVID-19 onset, per mo	NA	1.01 (1.00 to 1.02)	1.01 (1.00 to 1.01)	0.1^b^	.21	.003
Continuous after SB8 law change	NA	0.97 (0.95 to 0.99)	0.97 (0.96 to 0.99)	−0.5^b^	.001	.004
*Dobbs* decision						
Incident (July 2022)	NA	1.61 (1.39 to 1.87)	NA	10.7^c^	<.001	NA
Continuous after *Dobbs* decision, per mo	NA	1.10 (1.08 to 1.13)	1.07 (1.06 to 1.09)	1.7^b^	<.001	<.001
North Carolina 12-wk law change						
Incident (August 2023)	NA	0.70 (0.59 to 0.84)	NA	−9.8^c^	<.001	NA
Continuous after 12-wk law, per mo	NA	0.90 (0.87 to 0.92)	0.96 (0.94 to 0.98)	−0.9^b^	<.001	<.001
**Gestational length, wk**						
Continuous (since June 2016)	−0.02 (−0.03 to −0.01)	NA	−0.02 (−0.03 to −0.01)	−0.02^d^	<.001	NA
COVID-19 onset						
Incident (April 2020)	−0.74 (−1.09 to −0.40)	NA	NA	−0.7^e^	<.001	NA
Continuous after COVID-19 onset, per mo	0.05 (0.02 to 0.07)	NA	0.03 (0.01 to 0.05)	0.03^d^	.01	<.001
Continuous after SB8 law change, per mo	−0.11 (−0.18 to −0.04)	NA	−0.08 (−0.13 to −0.03)	−0.08^d^	.003	.002
*Dobbs* decision						
Incident (July 2022)	1.37 (0.91 to 1.83)	NA	NA	1.74^e^	<.001	NA
Continuous after *Dobbs* decision, per mo	0.45 (0.37 to 0.52)	NA	0.37 (0.31 to 0.42)	0.37^d^	<.001	<.001
North Carolina 12-wk law change						
Incident (August 2023)	−2.36 (−2.97 to −1.75)	NA	NA	−2.5^e^	<.001	NA
Continuous after 12-wk law, per mo	−0.55 (−0.63 to −0.47)	NA	−0.18 (−0.23 to −0.12)	−0.18^d^	<.001	<.001

Abbreviations: *Dobbs, Dobbs v Jackson Women’s Health Organization* decision; NA, not applicable; OR, odds ratio; SB8, Texas Senate Bill 8.

^a^ Slope between change points can be derived by summing β coefficients or multiplying ORs.

^b^ Change in percentage points per month.

^c^ Change in percentage points that month.

^d^ Change in weeks per month.

^e^ Change in weeks that month.

Results examining weeks’ gestation were consistent. Prior to *Dobbs*, the gestational length of DCAF callers’ pregnancies had been declining (coefficient, −0.08 [95% CI, −0.13 to −0.03] weeks’ gestation per month), reaching a low of an estimated mean gestational length of 9.8 (95% CI, 9.4-10.1) weeks the month prior to *Dobbs* (Figure 3 and Table 2). With the *Dobbs* decision, mean gestational length increased to 11.5 (95% CI, 11.3-11.8) weeks and continued to increase by 0.37 (95% CI, 0.31 to 0.42) weeks’ gestation per month, reaching a high of mean 15.9 (95% CI, 15.4-16.5) weeks’ gestation prior to the North Carolina 12-week law. The mean gestational length of callers’ pregnancies decreased to 13.4 (95% CI, 13.1-13.7) weeks the month following the North Carolina 12-week law; mean gestational length continued to decline thereafter (coefficient, −0.18 [95% CI, −0.23 to −0.12]). Results from all sensitivity models were unchanged.

## Discussion

This cross-sectional study of caller records from DCAF, a large regional abortion fund located adjacent to many of the states most impacted by post-*Dobbs* abortion bans, found immediate and lasting changes in the volume and gestational distribution of callers following the *Dobbs* decision and ensuing state abortion bans and restrictions. At *Dobbs*, there was a sudden reversal of a prior 6-year trend of increasing call volume to DCAF, as indicated by both an immediate sharp drop in callers and a continued drop every month thereafter for almost 1 year. This finding diverges from our hypotheses, informed by the results of 2 case studies of facilities in the Northwest, which found increases in patient volume post-*Dobbs*.^6,7^ This divergence suggests variation in how *Dobbs* affected patient volume at facilities vs abortion funds, as well as geographic variation in its impacts.

The decrease in DCAF callers over the months post-*Dobbs* occurred almost entirely among callers in their first trimester of pregnancy; the decline was far more gradual among callers at 13 weeks’ gestation or later. Thus, after *Dobbs*, there was a dramatic increase in the proportion of callers who were later in pregnancy (from approximately 22% just before *Dobbs* to >50% a year after *Dobbs*). In parallel, the mean gestational length of callers jumped immediately after *Dobbs* and continued to increase over the next 2 years.

One contributor to the decrease in first-trimester callers may be post-*Dobbs* innovations in abortion service delivery, such as telehealth and direct ordering from online pharmacies,^2^ both of which have lower price points than facility-based abortion care,^26^ potentially resulting in people seeking abortion during the first trimester having less need for financial support from DCAF. The outpouring of financial support to abortion funds throughout the country immediately following *Dobbs*^4,5^ may also have contributed to the reduced call volume to DCAF immediately post-*Dobbs*, as people seeking abortion had multiple sources of support. Additionally, this decrease may be the result of people seeking abortion forgoing pursuing care due to uncertainty about whether abortion was available during rapid legal change^27^ or of some people who might otherwise have sought abortion care continuing their pregnancies in the face of insurmountable barriers to abortion.^28^

Both before and after *Dobbs,* consistent with other research on abortion fund clients,^11,12,13,14,15^ DCAF callers were disproportionately seeking abortion care at or after 13 weeks. Although less than 10% of abortions nationally took place after the first trimester during the period leading up to *Dobbs*,^29^ the lowest proportion of people seeking abortions at 13 weeks or later in our study period was 22%, right before the *Dobbs* decision. People seeking abortion after 13 weeks’ gestation face growing barriers, including higher procedure cost^30^ and fewer practitioners,^31^ which can increase travel costs and logistical difficulties, potentially compounding to exacerbate delays in obtaining care. These factors increase the likelihood that people seeking abortions at or after 13 weeks’ gestation will need support from abortion funds, potentially explaining the steady volume of these callers.

What we cannot ascertain from these data is whether callers seeking later abortion care would have obtained a first-trimester abortion under pre-*Dobbs* service availability (and, post-*Dobbs*, were delayed into a later gestational length) or represent a concentration of people who needed later care as fewer facilities offered later care—or a combination of both. Research has documented long wait times for appointments following *Dobbs* as state bans went into effect^28^ and that the number of abortion facilities offering care at 13 weeks or later shrank.^31^ Having to wait for an appointment may have delayed some callers’ abortion timing, while reduced service availability nationally (and particularly in the South) may have compelled more people to seek abortion care in the DC area and request support from DCAF. Some callers may have experienced both factors.

One year after *Dobbs*, at the enactment of North Carolina’s 12-week ban, we saw a reversal in these trends: a large increase in callers, particularly first-trimester callers, and a drop in the proportion who were at 13 weeks’ gestation or later (vs first trimester). As noted, rapid policy changes can be confusing for people seeking abortion,^27^ and there may have been broad misunderstanding of first-trimester abortion’s continued legality in North Carolina.

These findings underscore that changes in abortion care and care-seeking associated with abortion bans and restrictions do not stop at state borders. Policy changes outside of the DC area were followed by large-scale and sudden changes in call volume at DCAF. They were also followed by an increase in caller mean gestational length, suggesting that changes in abortion legality have effects on the gestational distribution of abortion.

### Limitations

This study has important limitations. These results are not generalizable to other abortion funds that differ in geographic location, resources, relationships with abortion facilities, gestational limits on care provided, and caller volume. However, in the absence of systematic, large-scale, or reliable data collection efforts, we must rely on regional data for insight into the gestational distribution of abortions. Future research should examine caller data from other funds, as well as facility-based data. This study is also limited in its ability to account for additional exogenous factors that may have impacted caller volume and gestational length. Furthermore, this study is limited in its reliance on self-reported caller gestational length, which may not be accurate, although research demonstrates that pregnant people can reliably assess their gestational length.^32^ We also do not know callers’ gestational length at presentation for abortion, nor whether they proceeded to abortion. Because people seeking abortion later in pregnancy face substantial and compounding barriers that can extend the time until they obtain abortion care,^33^ our findings represent an underestimate of gestational length at time of abortion.

## Conclusions

In this cross-sectional study of more than 45 000 caller records from a large, uniquely situated abortion fund, we found that the *Dobbs* decision and subsequent abortion bans and restrictions were followed by a decline in the volume of callers seeking abortion assistance in the first trimester and a sharp and sustained increase in callers’ mean gestational length. Consistent with findings from other regional case studies,^6,7^ these results suggest a post-*Dobbs* differential redistribution of care-seeking by trimester and/or an increase in delay in presentation for care. These results can inform state policies and local practices to mitigate the effects of restrictive abortion laws on individuals needing abortion care.

## Notes

Supplement 1. **eFigure.** Flowchart of DCAF Caller Data, June 2016 to June 2024

Supplement 2.
Data Sharing Statement
