# Supplementary material for: Caller Volume and Gestational Length at an Abortion Fund After Dobbs
Source: JAMA Netw Open. 2025 Dec 3;8(12):e2546508. doi: 10.1001/jamanetworkopen.2025.46508 (PMC12676352; doi:10.1001/jamanetworkopen.2025.46508)
Supplement: Supplement 1. — eFigure. Flowchart of DCAF Caller Data, June 2016 to June 2024 [file jamanetwopen-e2546508-s001.pdf]

Supplemental Online Content

Kimport K, Schroeder R, Rocca CH. Caller volume and gestational length at an abortion fund after *Dobbs*. *JAMA Netw Open*. 2025;8(12):e2546508.
doi:10.1001/jamanetworkopen.2025.46508

eFigure. Flowchart of DCAF Caller Data, June 2016 to June 2024

This supplemental material has been provided by the authors to give readers additional information about their work.

© 2025 Kimport K et al. *JAMA Network Open*.

eFigure 1: Flowchart of DCAF Caller Data, June 2016 to June 2024

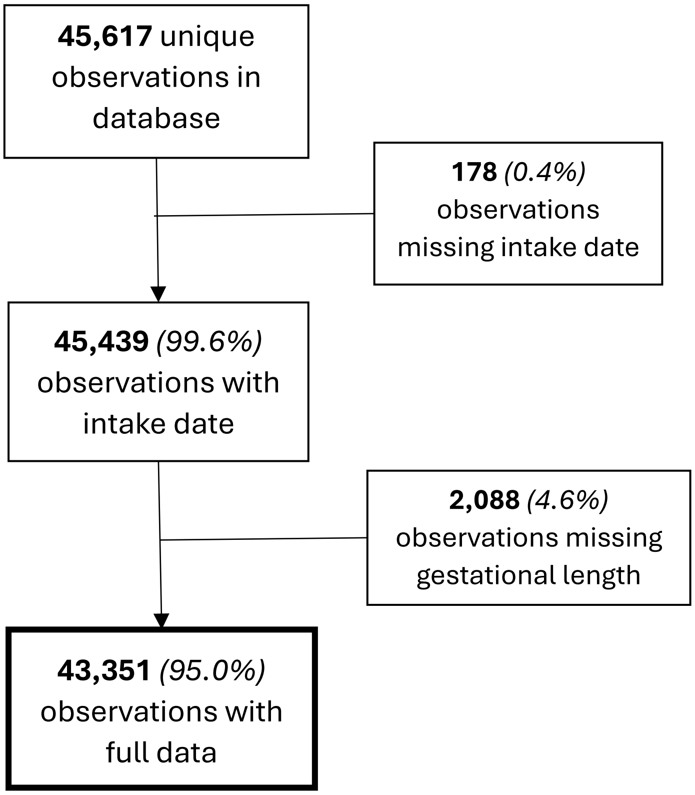

© 2025 Kimport K et al. *JAMA Network Open*.
