# Supplementary material for: Caller Volume and Gestational Length at an Abortion Fund After Dobbs
Source: JAMA Netw Open. 2025 Dec 3;8(12):e2546508. doi: 10.1001/jamanetworkopen.2025.46508 (PMC12676352; doi:10.1001/jamanetworkopen.2025.46508)
Supplement: Supplement 2. — Data Sharing Statement [file jamanetwopen-e2546508-s002.pdf]

Data Sharing Statement

Kimport. Caller Volume and Gestational Length at an Abortion Fund After Dobbs. *JAMA Netw Open*. Published December 03, 2025. doi:10.1001/jamanetworkopen.2025.46508

Data
Data available: No

Additional Information
Explanation for why data not available: Deidentified caller data were provided to the investigators by the District of Columbia Abortion Fund. Data cannot be shared publicly by investigators because they do not have permission to do so.
